# 100% oxygen mobilizes stem cells and up-regulates MIF and APRIL in humans: a new point on the hormetic dose curve

**DOI:** 10.3389/fcell.2024.1377203

**Published:** 2025-02-05

**Authors:** Kent J. MacLaughlin, Gregory P. Barton, Julia E. MacLaughlin, Jacob J. Lamers, Matthew D. Marcou, Matthew J. O’Brien, Rudolf K. Braun, Marlowe W. Eldridge

**Affiliations:** ^1^ Department of Pediatrics, University of Wisconsin, Madison, WI, United States; ^2^ Department of Internal Medicine, University of Texas Southwestern Medical Center, Dallas, TX, United States; ^3^ Medical Oxygen Hyperbaric Clinic, The American Center, Madison, WI, United States; ^4^ University of Wisconsin School of Medicine and Public Health, Madison, WI, United States

**Keywords:** oxygen therapy, stem cell mobilization and homing, inflammatory cyotokines, macrophage migration inhibition factor, hematopoietic (stem cell) transplant (HSCT), proangiogenic, A proliferation inducing ligand (APRIL), hemangioblast differentiation

## Abstract

**Introduction:**

The aim of the current study was to test normobaric 100% oxygen (NBO) (PiO2 = 713 mmHg) for stem cell mobilization and cytokine modulation. Although current oxygen therapy (PiO2 = 1,473–2,233 mmHg) is well known to mobilize stem cells and modulate cytokine, little is known about NBO and its place on the low dose stimulation phase of the hormetic dose curve of oxygen. We asked the question, will NBO mobilize stem cells and modulate cytokines. A positive outcome presents the potential to create and refine oxygen treatment protocols, expand access, and optimize patient outcomes.

**Methods:**

Healthy 30–35-year-old volunteers were exposed to 100% normobaric oxygen for 60 min, M-F, for 10 exposures over 2 weeks. Venous blood samples were collected at four time points: 1) prior to the first exposure (serving as the control for each subject), 2) immediately after the first exposure (to measure the acute effect), 3) immediately before the ninth exposure (to measure the chronic effect), and 4) three days after the final exposure (to assess durability). Blinded scientists used flow cytometry to gate and quantify the Stem Progenitor Cells (SPCs).

**Results:**

CD45dim/CD34+/CD133+ and CD45+/CD34+/CD133+ were significantly mobilized following nine daily one-hour exposures to normobaric 100% oxygen. Conversely CD45−/CD34+/CD133+, CD45-/CD34+/CD133− and CD45−/CD34−/CD133+ phenotypes were downregulated suggesting differentiation into more mature phenotypes. The CD133+ phenotype exhibited a maturing from CD45− to CD45dim stem cells. CD45−/CD34, CD45−/CD31 and CD45−/CD105 were downregulated with no changes in related CD45dim and CD45^+^ phenotypes. The cytokines “macrophage migration inhibitory factor” (MIF) and “a proliferation inducing ligand” (APRIL) were significantly upregulated.

**Conclusion:**

This study demonstrates that 100% normobaric oxygen mobilizes stem cells and upregulates the expression of the inflammatory cytokines marking a new point on the low dose stimulation phase of the hormetic dose curve of oxygen.

## Introduction

Since the first use of oxygen for respiratory support in 1885, the utility of oxygen has continually evolved concurrently with the evolution of our understanding of the mechanisms and biological effects of oxygen dose. One of these biological effects, stem cell mobilization, provided a critical mechanism on the role that cellular oxygen tension plays in tissue healing and regeneration (Thom et al., 2006). Subsequent research established a direct relationship between oxygen dose and stem cell mobilization (Heyboer et al., 2014). The mechanism of stem cell mobilization by oxygen dose is increased nitric oxide in the bone marrow (Goldstein et al., 2006) resulting in accelerated blood vessel formation and wound healing (Gallagher et al., 2006; Milovanova et al., 2008). These papers established two points on the dose stimulation phase of the hormetic dose curve of oxygen to be at 2.0 atm absolute breathing 100% oxygen (PiO2 = 1,426 mmHg) and 2.4 atm absolute breathing 100% oxygen (PiO2 = 1,777 mmHg).

The low dose stimulation phase of the hormetic dose curve of oxygen, however, has not been fully elucidated. The minimal dose required to initiate stem cell mobilization and cytokine modulation was first investigated in 2018 in an experiment done in our lab. This experiment demonstrated that stem cells are mobilized by 42% normobaric oxygen (PiO2 = 300 mmHg) in a rat model (MacLaughlin et al., 2019). A subsequent experiment also done in our lab in 2022 established a new low dose stimulation point of 1.27 atm absolute hyperbaric air (PiO2 = 189 mmHg). That investigation resulted in mobilized stem progenitor cells (SPCs) by two-fold following nine exposures to 1.27 ATA hyperbaric air, further increasing to a three-fold increase 72 h post the tenth exposure, indicating not only an immediate but also a durable effect (MacLaughlin et al., 2023).

In an effort to further elucidate the low dose stimulation phase of the hormetic dose curve of oxygen, in the present experiment we test NBO (100% normobaric medical oxygen) (PiO2 = 713 mmHg) for stem cell mobilization and inflammatory cytokine modulation.

The COVID-19 pandemic at first overwhelmed the manufacture and supply channels of oxygen, but eventually resulted in improvements and as a result its world wide availability has increased (Organization, 2021). Although oxygen was used during the COVID-19 pandemic mainly for its ability to provide supplemental oxygen to the lungs helping to maintain adequate blood oxygen levels, it was unknown if there were other mechanisms involved (i.e., stem cell mobilization and cytokine modulation).

Recent studies have demonstrated that relatively low oxygen tensions (PiO2’s) can yield significant biological responses (MacLaughlin et al., 2019; MacLaughlin et al., 2023; Miller et al., 2015; Cifu et al., 2014). These findings support the idea that low oxygen levels can substantially impact stem cell dynamics and inflammatory processes. The current study aims to build upon this knowledge by examining the effects of a nearly 5-fold increase in oxygen partial pressure within a normobaric setting, potentially informing the standard of care by demonstrating significant biological responses to NBO, hypothetically refining treatment protocols and optimizing patient outcomes.

The results from the present study may also help to understand a recent case report of normobaric 100% oxygen effectively ameliorating the anoxic injury following near drowning. The two-year-old child, who exhibited no speech, gait, or responsiveness to commands on the 48th day post-hospital discharge, showed remarkable improvement following daily treatments of normobaric 100% oxygen (administered at 2 L/min for 45 min via nasal cannula, twice daily). The treatment was administered over a period of 23 days, after which the patient’s condition had stabilized sufficiently to allow for transfer to a hyperbaric oxygen therapy center. There, the patient made a very substantial recovery (Harch and Fogarty, 2017).

We asked the question: Will 100% oxygen, without added hyperbaric pressure, mobilize stem cells and modulate inflammatory cytokines? We hypothesized that it would do both.

## Materials and methods

### Design and subjects

This study is a prospective, randomized, single-blind study conducted at the University of Wisconsin–Madison Clinical Sciences Center between 1 May 2021, and 31 August 2021. This study was approved by the Institutional Review Board of the University of Wisconsin–Madison under UW IRB ID: 2020-0293-CR001. All participants provided written informed consent. We recruited healthy adults 35 and under for participation in this study. Six (6) women and eight (8) men participated in this study (n = 14). Five subjects dropped out of the study prior to completion (Table 1).

**TABLE 1:** Anthropometric data of subjects in this 100% oxygen concentration study.

	N	Mean	Standard deviation
Age	14	32.86 years	3.7 years
Height	14	173.79 cm	7.0 cm
Weight	14	77.62 kg	12.7 kg
BMI	14	25.26%	2.8%

Exclusions included the following; Standard contraindications to Hyperbaric Oxygen, pregnancy, nicotine use/addiction, active smoking or vaping within 1 month, inability to equalize inner ear pressure, acute hypoglycemia, uncontrolled diabetes mellitus, confinement anxiety, previous barotrauma, pregnant or intending to become pregnant, or currently nursing, medical or psychological conditions which might create undue risk to the subject or interfere with the subject’s ability to comply with the protocol requirements, diagnosed sleep apnea, previous or active COVID-19 infection, emphysema with CO2 retention, subjects with “high” exercise scores using the Global Exercise Score index.

#### Normobaric 100% oxygen concentration exposure

Subjects breathed normobaric 100% medical grade oxygen (>99%) (AirGas Products–Radnor, PA) through a tight-fitting mask with two one-way non-rebreather valves (Hans Rudolph, Shawnee, Kansas) attached. Subjects visited the lab 11 times over the course of 15 days for exposure and/or sample collection. We accounted for circadian cell cycle variations including acrophase by providing all exposures and collecting all blood samples at the same time of day over the 15-day experiment. All subjects were diurnally active.

#### Sample collection

**FIGURE 1:**
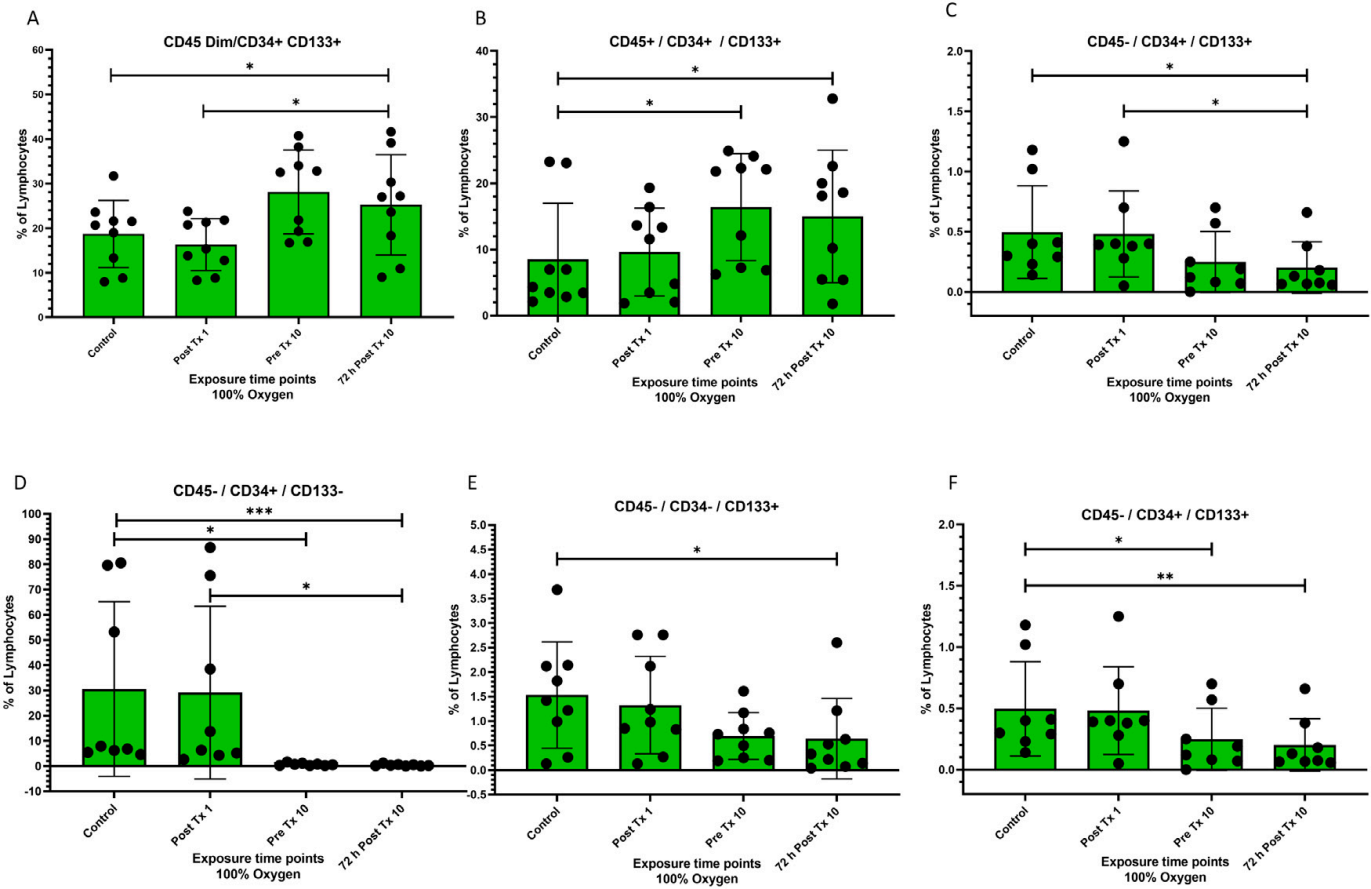
Frequency of stem cell phenotypes at four time points after 100% oxygen exposure detected by flow cytometry. Control = immediately prior to first exposure, post t × 1 = immediately after the first exposure, Pre T × 10 = immediately prior to exposure 10, 72 h Post T × 10 = 3 days after final exposure 10. **(A)** CD45dim/CD34+/CD133+ **(B)** CD45+/CD34+/CD133+, **(C)** CD45−/CD34+/CD133+ **(D)** CD45−/CD34+/CD133− **(E)** CD45−/CD34−/CD133+ **(F)** CD45−/CD34−/CD133−.

A 21- or 23-gauge BD Vacutainer Safety-Lok Blood Collection Set (Becton, Dickinson and Company, Franklin Lakes, NJ United States) into a Cyto-Chex BCT tube (Streck Inc NE United States) was used to collect peripheral venous blood. Collected blood was stored according to the manufacturer’s instructions. Venous blood samples were collected with subjects sitting or supine. The study protocol in graphic format is included in Figure 1.

#### Flow cytometry

Flow cytometry was performed as previously described (MacLaughlin et al., 2023). All samples were prepared for flow cytometry by placing prepared human samples into flow cytometry tubes appropriately labeled for fluorescence minus one (FMO) tubes, rainbow bead tubes, and single antibody tubes to be used as gating references. Antibodies were pipetted into flow cytometry tubes according to the manufacturer’s instructions. Antibodies used include CD34 = Brilliant Violet 421 (BioLegend San Diego, CA United States), CD45 = Alexa Fluor 488 (BioLegend San Diego, CA United States), CD133 = PE (Miltenyi Biotec, North Rhine-Westphalia, Germany) CD31 = Brilliant Violet 605 (BioLegend San Diego, CA United States), CD105 = PE-Cy7 (BioLegend San Diego, CA United States), Ghost Dye Red 780 = Tonbo Biosciences, San Diego, CA.

A ThermoFisher Attune NxT (Waltham, MA United States) for Flow cytometry and FlowJo software (FlowJo, Ashland, OR, United States) was used to gate the results. All gating was performed by blinded scientists at the University of Wisconsin Carbone Cancer Laboratory (Madison, WI United States). Lymphocytes were gated by forward and side scatter and doublets were excluded. CD45 positive, negative, and dim cells were selected for further analysis of the expressions of CD34, CD133, CD105, and CD31.

### Enzyme-linked immunosorbent assay

As previously described the Invitrogen ProcartaPlex™ Human Immune Monitoring Panel 65-Plex (Invitrogen, Waltham, MA, United States) was utilized to evaluate changes in cytokines, chemokines, and growth factors. The Invitrogen ProcartaPlex™ Human Immune Monitoring Panel 65-Plex includes tests for a wide range of cytokines, chemokines, and growth factors using sandwich ELISA principles with two specific antibodies binding to different epitopes of a protein, allowing simultaneous quantitation of all protein targets with a Luminex instrument. The targets analyzed are listed below:

Cytokines: G-CSF, GM-CSF, IFN alpha, IFN gamma, IL-1 alpha, IL-1 beta, IL-2, IL-3, IL-4, IL-5, IL-6, IL-7, IL-8 (CXCL8), IL-9, IL-10, IL-12p70, IL-13, IL-15, IL-16, IL-17A, IL-18, IL-20, IL-21, IL-22, IL-23, IL-27, IL-31, LIF, M-CSF, MIF, TNF alpha, TNF beta, TSLP.

Chemokines: BLC (CXCL13), ENA-78 (CXCL5), Eotaxin (CCL11), Eotaxin-2 (CCL24), Eotaxin-3 (CCL26), Fractalkine (CX3CL1), Gro-alpha (CXCL1), IP-10 (CXCL10), I-TAC (CXCL11), MCP-1 (CCL2), MCP-2 (CCL8), MCP-3 (CCL7), MDC (CCL22), MIG (CXCL9), MIP-1 alpha (CCL3), MIP-1 beta (CCL4), MIP-3 alpha (CCL20), SDF-1 alpha (CXCL12).

Growth Factors/Regulators: FGF-2, HGF, MMP-1, NGF beta, SCF, VEGF-A.

Soluble Receptors: APRIL, BAFF, CD30, CD40L (CD154), IL-2R (CD25), TNF-RII, TRAIL (CD253), TWEAK.

This panel allows for the simultaneous analysis of these 65 protein targets, providing a comprehensive profile of immune responses2. All tests were conducted by blinded scientists at the University of Wisconsin Non-Human Primate Research Center (Madison, WI, United States).

### Statistical analysis

All statistical analyses were calculated using Graph Pad Prism 9.0.0 (GraphPad Software, San Diego, CA, United States). All calculations utilized the Wilcoxon matched-pairs signed-rank test. Comparisons between all-time points were performed. *A priori* at the 0.05 level was used to indicate the significance level and all tests were 2-tailed. Statistical analyses were calculated using Graph Pad Prism (GraphPad Prism 9.0.0 Software, San Diego, CA, United States).

## Results

In this study 9 humans were exposed to normobaric 100% oxygen (NBO) 10 times (Monday-Friday) over the course of 15 days. To determine whether stem progenitor cells (SPCs) are mobilized by normobaric 100% oxygen concentration, blinded scientists used flow cytometry to survey SPC expression using Clusters of Differentiation (CD) cell surface markers at 4 time points. Blinded scientists also used the Invitrogen ProcartaPlex™ Human Immune Monitoring Panel 65-Plex (Invitrogen, Waltham, MA, United States) to evaluate changes in cytokines, chemokines, and growth factors.

### CD34/CD133

Both CD45^dim^/CD34^+^/CD133^+^ and CD45+/CD34+/CD133+ were significantly mobilized just prior to the 10th exposure of normobaric 100% oxygen exposures (*p* = 0.02) (Figures 1A, B respectively), while the frequency of CD45^−^/CD34^+^/CD133^+^ was significantly decreased just prior to the 10th exposure to normobaric 100% oxygen exposures (*p* = 0.02) (Figure 1C). CD45^−^/CD34^+^/CD133^−^, CD45^−^/CD34^−^/CD133^+^, and CD45^−^/CD34^+^/CD133^+^ were significantly decreased (Figures 1D–F respectively).

### CD133

**FIGURE 2:**
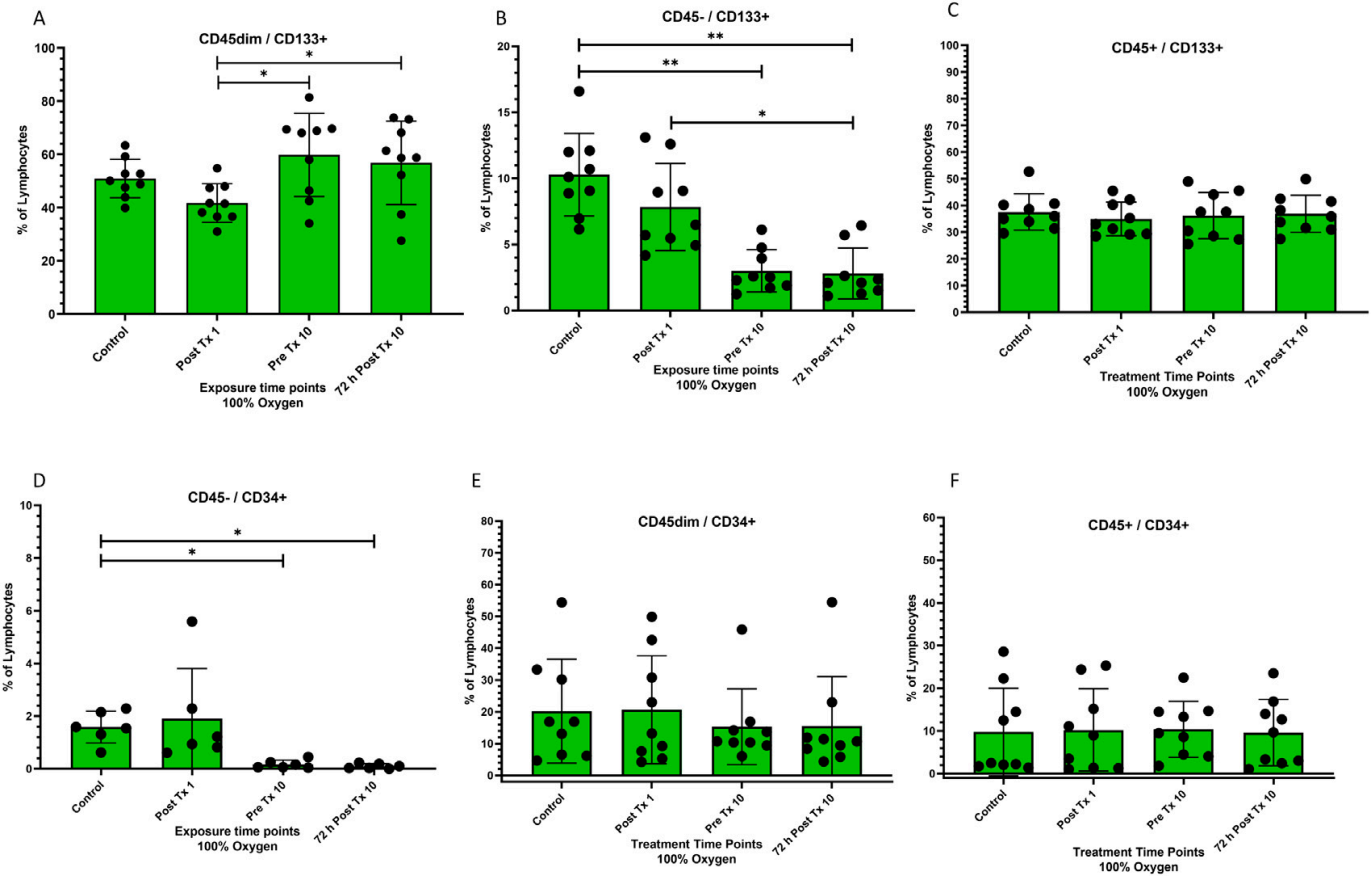
Frequency of stem cell phenotypes at four time points after 100% oxygen exposure detected by flow cytometry. Control = immediately prior to first exposure, post t × 1 = immediately after the first exposure, Pre T × 10 = immediately prior to exposure 10, 72 h Post T × 10 = 3 days after final exposure 10. **(A)** CD45dim/CD133+ **(B)** CD45−/CD133+, **(C)** CD45+/CD133+ **(D)** CD45−/CD34+ **(E)** CD45dim/CD34+ **(F)** CD45+/CD34+.

CD45^dim^/CD133^+^ stem cells were significantly mobilized between the end of the first exposure and just prior to the 10th exposure (p = 0.02) and remained significantly increased for 72 h following the 10th and final exposure (p = 0.03) while CD45^−^/CD133^+^ stem cells decreased significantly (p = 0.004) and remained decreased for 72 h following the 10th and final exposure (*p* = 0.0008). CD45^+^/CD133^+^ was unchanged (Figures 2A–C respectively).

### CD34

CD45^−^/CD34^+^ decreased significantly prior to the 10th exposure (p = 0.03) and remained decreased for 72 h after the 10 and final exposure (p = 0.03) (Figure 2D). CD45^dim^/CD34^+^ and CD45^+^/CD34^+^ stem cells remained unchanged (Figures 2E, F respectively).

### CD31

**FIGURE 3:**
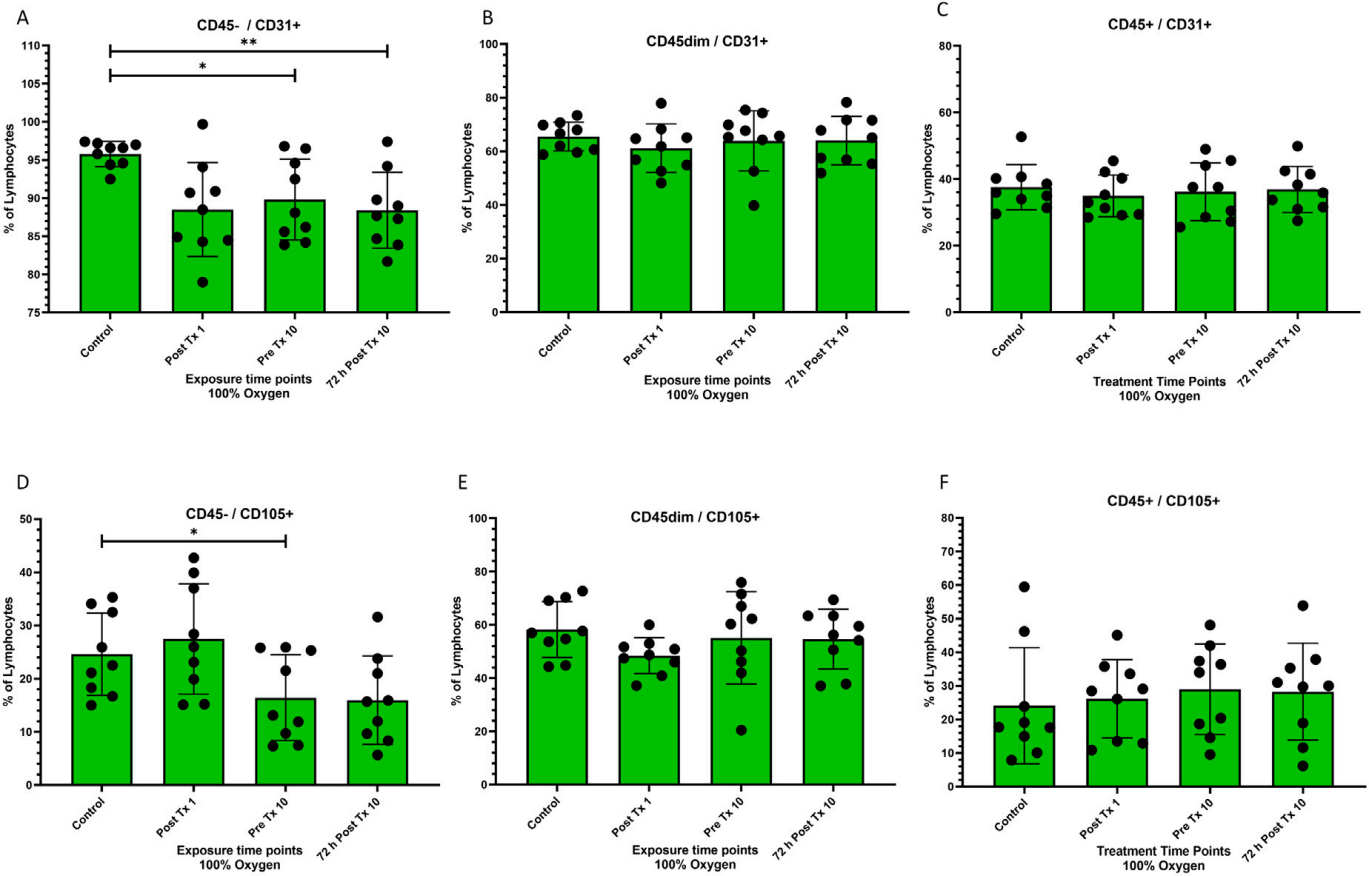
Frequency of stem cell phenotypes at four time points after 100% oxygen exposure detected by flow cytometry. Control = immediately prior to first exposure, post t × 1 = immediately after the first exposure, Pre T × 10 = immediately prior to exposure 10, 72 h Post T × 10 = 3 days after final exposure 10. **(A)** CD45−/CD31+ **(B)** CD45dim/CD31+, **(C)** CD45+/CD31+ **(D)** CD45−/CD105+ **(E)** CD45dim/CD105+ **(F)** CD45+/CD105+.

CD45^−^/CD31^+^ decreased significantly prior to the 10th exposure (*p* = 0.04) and remained decreased for 72 h after the 10 and final exposure (p = 0.008) (Figure 3A). CD45^dim^/CD31^+^ and CD45^+^/CD31^+^ stem cells remained unchanged (Figures 3B, C respectively).

### CD105

CD45^−^/CD105^+^ decreased significantly prior to the 10th exposure (*p* = 0.04) (Figure 3D). CD45^dim^/CD105^+^ and CD45^+^/CD105^+^ stem cells remained unchanged (Figures 3E, F respectively).

### A proliferation-inducing ligand (APRIL)

**FIGURE 4:**
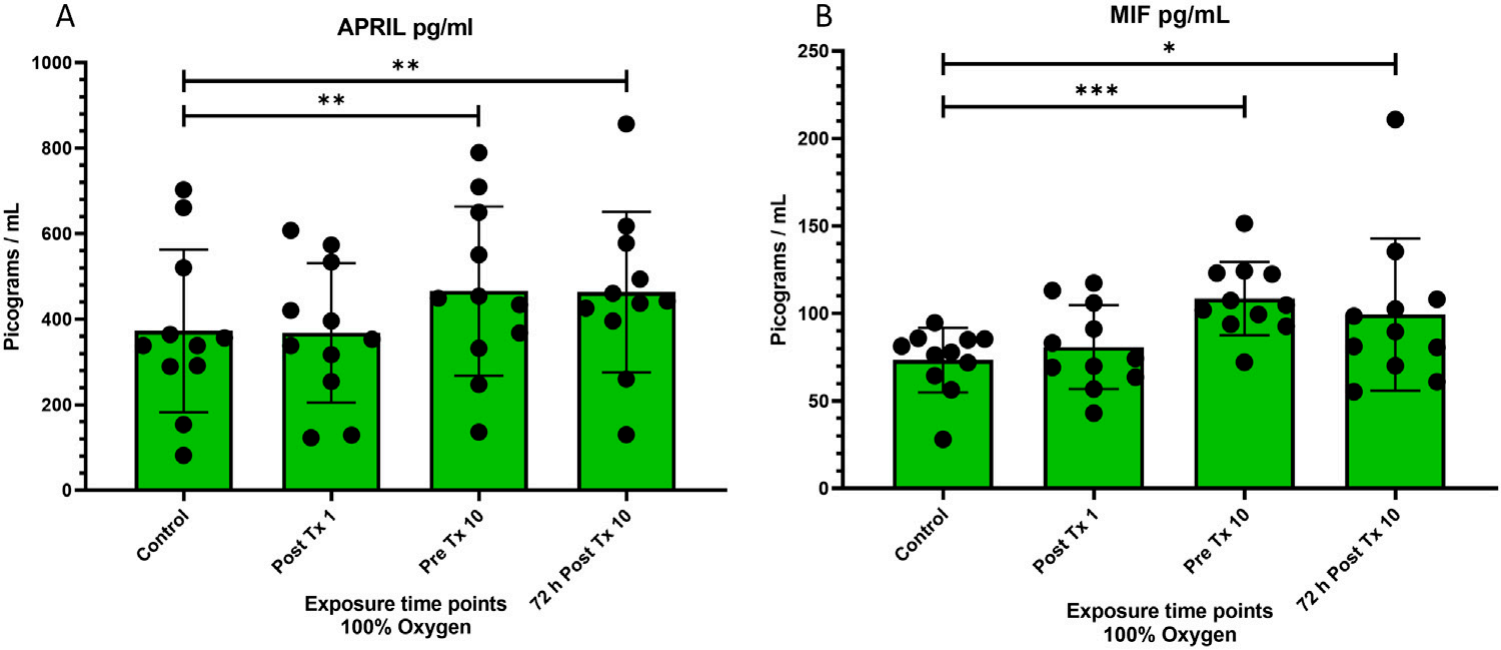
Levels of “a proliferation inducing ligand” (APRIL or TNFSF13) and “macrophage migration inhibitory factor” (MIF) at four time points after 100% oxygen exposure. Control = immediately prior to first exposure, post t × 1 = immediately after the first exposure, Pre T × 10 = immediately prior to exposure 10, 72 h Post T × 10 = 3 days after final exposure 10. **(A)** a proliferation-inducing ligand (APRIL or TNFSF13) **(B)** macrophage migration inhibitory factor (MIF).

A Proliferation-Inducing Ligand was very significantly increased prior to the 10th exposure (p = 0.007) and remained significantly increased for 72 h following the 10 and final exposure (p = 0.004) (Figure 4A).

### Macrophage migration inhibitory factor (MIF)

Macrophage migration inhibitory factor significantly increased prior to the 10th exposure (p = 0.001) and remained significantly increased for 72 h following the 10 and final exposure (p = 0.04) (Figure 4B).

## Discussion

It is well established that Hyperbaric oxygen therapy mobilizes stem cells (Thom et al., 2006; Heyboer et al., 2014; Thom et al., 2011) and modulates inflammatory cytokines (Hedetoft et al., 2021; Benson et al., 2003; Feng et al., 2016). Stem cells are mobilized by hyperbaric oxygen therapy at 2.0 atm absolute (PiO2 = 1,426 mmHg) and 2.4 atm absolute (PiO2 = 1,711 mmHg) in a dose dependent manner (Heyboer et al., 2014).

Conversely, it is unknown if normobaric 100% oxygen therapy with a PiO2 of 713 mmHg will mobilize stem cells or modulate cytokine expression.

We previously demonstrated that 1.27 ATA hyperbaric air with increased barometric pressure only (PiO2 = 190 mmHg) mobilized stem cells and modulated cytokines in humans (MacLaughlin et al., 2023). We also demonstrated that a 42% oxygen concentration without increased barometric pressure (PiO2 = 299 mmHg) did the same in a rat model (MacLaughlin et al., 2019).

In the present investigation, we tested 100% oxygen concentration without added barometric pressure (PiO2 = 713 mmHg) in humans.

We asked the question, will daily exposures to normobaric 100% oxygen mobilize stem cells and modulate cytokines in humans? We used a randomized, single-blind study design to test this question. Our hypothesis proposed that stem cells would be mobilized and inflammatory cytokines would be modulated. Our findings did indeed demonstrate significant stem cell mobilization/differentiation and cytokine modulation. These findings suggest a new point on the low dose stimulation phase of the hormetic dose curve of oxygen.

This study provides a new perspective on oxygen dose, mechanism and function. Normobaric 100% oxygen potentially provides a viable option for many pathologies including hematopoietic stem cell repopulation and conditions associated with diminished endothelial function.

The increase in the inflammatory cytokines MIF and APRIL was unexpected and the implications are intriguing. APRIL is crucial to maintain and proliferate some B cells and plasmacytes. MIF is essential in the tissue repair and regeneration process and has been shown to promote the regeneration of peripheral nerves resulting in beneficial axonal growth.

### Critical analysis and major findings

In this experiment, blinded scientists utilized flow cytometry to profile stem cell mobilization and cytokine expression in four chronological peripheral venous blood samples, The major finding of this study is the novel demonstration that daily 60-minute exposures to normobaric 100% oxygen mobilizes and differentiates stem cells and modulates inflammatory cytokines in humans. Following ten consecutive weekdays of daily 60-minute exposures, we observed a significant mobilization of CD45dim/CD34+/CD133+, and CD45+/CD34+/CD133+ stem progenitor cells (SPCs) with corresponding reductions in the populations of CD45−/CD34+/CD133+, CD45−/CD34+/CD133−, and CD45−/CD34-/CD133+ and no change in CD45−/CD34−/CD133− (Figure 1A through Figure 1F respectively). The mobilization of CD45dim/CD34+/CD133+ cells coupled with reductions in CD45−/CD34+/CD133+, CD45−/CD34+/CD133−, and CD45−/CD34−/CD133+, may hypothetically be attributed to the differentiation of primitive CD133+ hemangioblast cells into more mature CD34^+^ proangiogenic progenitor cells (Friedrich et al., 2006) occurring simultaneously with the progression of CD45− to CD45dim. Other hypothetical mechanisms may explain these outcomes, including homing of CD45dim/CD34+/CD133− stem cells to sites of endothelial repair and angiogenesis (Van Craenenbroeck et al., 2013; Kwon et al., 2014) or differentiation of CD45dim/CD34−/CD133+ cells into hematopoietic (Gordon et al., 2003; Miraglia et al., 1997) or endothelial cells (Gehling et al., 2000).

The mechanism responsible for mobilizing SPCs in this experiment is beyond the scope of this study but may be similar to that found in higher dose oxygen therapy (HBOT), which activates nitric oxide synthase and plays a key role in initiating SPC mobilization (Aicher et al., 2003; Thom and Buerk, 2003; Thom et al., 2003). Cellular oxygen partial pressure regulates Hypoxia-Inducible Factor 1 (HIF1), which consists of HIF1-α and HIF1-β subunits (Forsythe et al., 1996; Jiang et al., 1996; Semenza et al., 1997; Semenza, 1999; Semenza, 2020; Sunkari et al., 2015). A prominent theory, known as the Hyperoxic Hypoxic Paradox (HHP) or the Normobaric Oxygen Paradox (NOP), suggests that hyperoxia may stabilize HIF proteins through the induction of endogenous antioxidants (Salvagno et al., 2022; Fratantonio et al., 2021; Balestra et al., 1985; Burk, 2007; Hadanny and Efrati, 2020; De Bels et al., 2012; Rocco et al., 2014). The HHP/NOP begins with an elevation in cellular oxygen tension resulting from breathing an increased partial pressure of oxygen, leading to a rise in reactive oxygen species (ROS) as a result of oxygen metabolism within the mitochondrial electron transport complex subunits (Brueckl et al., 2006; Waypa et al., 2016). The increase in ROS leads to hormetic stress, which upregulates the production of endogenous antioxidants (Godman et al., 2010). The endogenous antioxidants persist for several hours (Suzuki et al., 2008; Broeyer et al., 2008) and accumulate in the cell. Upon return to normoxic conditions following hyperoxic exposure, the heightened levels of endogenous antioxidants reduce the number of ROS molecules lower than what is found in typical normoxic conditions. This results in a relative hypoxia at the cellular level, thereby stabilizing HIF1α. Stabilized HIF1α then translocates to the nucleus, where it dimerizes with the HIF1-β subunit to form the HIF complex, initiating downstream genetic transcription (Semenza, 2001). Leading to the activation of eNOS in the bone marrow parenchyma (Goldstein et al., 2006; Aicher et al., 2003; Thom et al., 2003; Thom et al., 2002) and stem cell mobilization.

CD45^dim^/CD34−/CD133+ cells are primitive blast and stem cells with the ability to differentiate into multiple cell lines including CD45^dim^/CD34+/CD133+ and CD45^dim^/CD34+/CD133−. These primitive stem cells are functionally potent with respect to homing and vascular repair (Friedrich et al., 2006).

CD45^dim^/CD34+/CD133+ stem cells are less primitive than CD45−/CD34−/CD133+ stem cells. The increase in CD45^dim^/CD34+/CD133+ stem cells (Figure 1A) found in our data may be related to the differentiation from CD45−/CD34−/CD133+ stem cells (Figure 1E) as a progression toward maturation and hypothetical endothelial repair although other explanations exist. A decrease in CD45^dim^/CD34+/CD133+ has been inversely correlated with both aging and chronic heart failure (CHF) (Fritzenwanger et al., 2009). Both experimental and clinical data support this (Andreou et al., 2006). The increase in CD45^dim^/CD34+/CD133+ (Figure 1A) following normobaric 100% oxygen exposures holds promise for both CHF and managing the progression of executive function, cognitive decline, Alzheimer’s and dementia, and possibly other diseases that correlate with decreased endothelial function.

### CD133

We also found a significant increase in CD45dim/CD133+ stem cells between the first and 10th exposure, with a corresponding decrease in CD45-/CD133+ and no change in CD45+/CD133+ (Figures 2A–C respectively). One explanation may be that CD45dim/CD133+ SPC’s (Figure 2A) were mobilized from the bone marrow stroma following repeated NBO exposures but it is also possible that CD45-/133+ (Figure 2B) cells matured or differentiated into CD45dim/CD133+ SPC’s leading to a decrease in the former and an increase in the latter. CD45^dim^/CD133+ are primitive stem progenitor cells expressed on hematopoietic (Gordon et al., 2003), endothelial (Gehling et al., 2000) and neural stem cells (Uchida et al., 2000). They are capable of differentiation into hematopoietic and endothelial cells and are sometimes classified as endothelial progenitor cells (EPC’s) depending on downstream differentiation. CD133+ cells originate in the bone marrow (Goldstein et al., 2006; Gallagher et al., 2006) and are involved in hematopoiesis, wound healing, and endogenous endothelial repair. They can proliferate, migrate and differentiate into several cell phenotypes (Luttun et al., 2002; Szmitko et al., 2003) and can also be angiogenic (Prater et al., 2007).

### CD34

The CD45-/CD34+ population was significantly reduced to almost zero while both CD45dim/CD34+ and CD45+/CD34+ were unchanged (Figures 2D–F respectively). This progression did not follow the pattern previously exhibited in CD45/CD133+ stem cells. The fate of the CD45−/CD34+ cells (Figure 2D) remains unclear but may be attributed to differentiation into another hematopoietic cell phenotype (Anjos-Afonso and Bonnet, 2023) or participation in angiogenesis (Siemerink et al., 2012).

Adult stem cells are a group of specific cell phenotypes that possess the abilities of self-renewal, multipotent differentiation, and repair after injury. Breathing 100% oxygen at hyperbaric levels activates nitric oxide synthase which plays a prime role in initiating CD34+ SPC mobilization (Goldstein et al., 2006; Aicher et al., 2003; Thom et al., 2003; Thom et al., 2002). CD133+ are hematopoietic precursors to CD34+ and almost all hematopoietic pluripotent and committed stem cells in colony-forming assays express CD34+ (Faramarz et al., 2018). In this study, we found that CD45dim/CD133+ SPCs were mobilized while at the same time, CD45dim/CD34+ SPCs were unchanged. We hypothesize that the exposure to intermittent hyperbaric air may mobilize CD133+ from bone marrow and also play a role in the differentiation of the CD133+ primitive hematopoietic precursor into mature hematopoietic cells or remain unchanged in their primitive state. It is also possible that CD45−/CD133+ cells differentiated into CD45dim/CD133+ SPC’s in response to repeated NBO exposures.

### CD31+

CD31 responded to normobaric oxygen (NBO) similarly to CD34, with a significant reduction in the CD45− phenotype and no change in CD45dim and CD45+ phenotypes (Figure 3A through Figure 3C respectively). Although the changes in CD31+ are beyond the scope of this investigation, it is plausible that they may have participated in angiogenesis (Yang et al., 1999). It is not surprising that CD31+ was increased by NBO as CD31+ is known to be mobilized by hyperbaric oxygen (Chen et al., 2021). The CD31 phenotype is known to participate in angiogenesis and is a therapeutic target in experimental treatments for atherosclerosis (Caligiuri, 2020).

### CD105+

CD105 like CD34 and CD31 responded to NBO with a downregulation in the expression of the CD45− phenotype and no change in either the CD45dim or CD45+ phenotypes (Figure 3D through Figure 3F respectively). CD105 is also associated with angiogenesis (Nassiri et al., 2011) and is more highly expressed in micro-vessels of diabetic patients (Fukumitsu et al., 2013). Although it is beyond the scope of this project to determine the outcome of CD105 phenotypes, these novel findings may be worth further investigation.

### A proliferation-inducing ligand (APRIL)

A proliferation inducing Ligand (APRIL) was significantly upregulated over the course of 9 days of NBO (p = 0.007) and remained significantly increased for 72 h after the final exposure (P = 0.004). This novel finding is important as it may help develop the genetic and physiological cascade resulting in stem cell mobilization following oxygen therapy.

APRIL, which is also known as tumor necrosis factor ligand superfamily member 13 (TNFSF13) and CD256, APRIL is a protein that is part of the tumor necrosis factor (TNF) family. TNF family members induce pleiotropic biological responses, including cell differentiation, growth, and apoptosis. APRIL is involved in the regulation of immune responses and is crucial in long-term survival of plasma cells in the bone marrow (Belnoue et al., 2008), in the development and survival of bone marrow derived B-cells and acts as a co-stimulator in the proliferation of B and T cells (Stein et al., 2002).

APRIL is also involved in the production of inflammatory cytokines and chemokines (van Deventer, 1999). However its importance in the proliferation and survival of a subset of B cells has been shown to have an anti-inflammatory effect (Kumar and Axtell, 2023), exposing a nuanced dual role both inflammatory and ant-inflammatory.

In a 2020 study, whole exome sequencing was completed on primary cells and plasma in one patient with Common Variable Immunodeficiency analyzing TNFSF13 mRNA expression *in vitro* using flow cytometry and next-generation sequencing. Results indicated that APRIL mRNA were completely absent in the monocytes and iPSC-moDCs of the patient (Yeh et al., 2020). A deficiency in APRIL can significantly impact the human immune system. APRIL is crucial for the maintenance of plasmacytes, which are cells responsible for producing immunoglobulins (antibodies). Without sufficient APRIL, the development and maintenance of these plasmacytes are disrupted, leading to a condition known as common variable immunodeficiency (CVID). This condition is characterized by an increased susceptibility to infections due to reduced levels of immunoglobulins.

### Macrophage migration inhibitory factor (MIF)

Macrophage migration inhibitory factor (MIF) was significantly upregulated over the course of 9 days of NBO (p = 0.007) and remained significantly increased for 72 h after the final exposure (P = 0.004). This novel finding, like the similar finding of increased expression of APRIL following oxygen therapy, may be an important piece of the puzzle that leads to stem cell mobilization following oxygen therapy. These cytokine findings are interesting subjects for future investigations.

MIF is an inflammatory cytokine that plays a critical role as a regulator of innate immunity by inhibiting the random movement of macrophages and promoting their accumulation at sites of inflammation and triggering various immune responses.

MIF regulates the immune response and is associated with many diseases including autoimmune diseases, cancer, metabolic disorders, and sepsis. MIF also regulates adaptive immune responses. MIF is released by immune cells and activated leukocytes and binds to CD74 receptors on other immune system cells. MIF is also known as glycosylation-inhibiting factor (GIF), phenylpyruvate tautomerase, and L-dopachrome isomerase.

MIF is known for its proinflammatory effects and is also implicated in the reparative process. Combined with CD74, MIF has been shown to promote wound healing in inflammatory bowel disease (Farr et al., 2020). Most interestingly MIF signaling via CD74 triggered the proliferation and differentiation of progenitor cells into epithelial like cells in the lung possibly participating in the alveolar barrier restoration (Marsh et al., 2009).

Very importantly the inflammatory response is one of most significant biological processes following sciatic nerve injury. MIF has been shown to promote the regeneration of peripheral nerves and Schawnn cells by inducing an inflammatory state in the Schawnn cells via CD74 receptor, providing beneficial axonal regrowth (Nishio et al., 2002; Song et al., 2019). These studies challenge the simplistic view that MIF is solely a pro-inflammatory or anti-inflammatory cytokine. Instead, they suggest that MIF may play a dual role, both contributing to tissue damage and aiding in injury repair.

The increase in both APRIL and MIF was unexpected as HBOT, a much higher dose of oxygen including an increase in barometric pressure, is known to have anti-inflammatory effects (Wang et al., 2024; Vlodavsky et al., 2006; Mrakic-Sposta et al., 2023; Capó et al., 2023; Lippert and Borlongan, 2019).

### Limitations

Our study is limited by a relatively small sample size. We were also limited to 10 hyperbaric air exposures in this experiment due to COVID-19 restrictions. This study should be repeated increasing the sample size and the exposures to 40 which is the standard number of exposures used in hyperbaric studies. We hypothesize that increasing the number of exposures will result in an increased number of statistically significant findings and will increase the magnitude of significance in existing findings. This study was also limited by a relatively small age range and the lack of a pathology. Future research should include expanded age range and include a specific pathology.

### Conclusion and impact

In this study, we demonstrate for the first time, that daily exposures to normobaric 100% Oxygen mobilize and differentiate proangiogenic and hematopoietic stem cell phenotypes and modulates inflammatory cytokines. These novel findings have many implications in the field of medical gas therapy. Although leading oxygen therapy medical societies have not included normobaric 100% oxygen in their repertoires at this time, these findings lay the foundational knowledge for them to include NBO in the future.

## Data Availability

The raw data supporting the conclusions of this article will be made available by the authors, without undue reservation.
